# Genetic polymorphisms of C-type lectin receptors in Behcet’s disease in a Chinese Han population

**DOI:** 10.1038/s41598-017-05877-x

**Published:** 2017-07-13

**Authors:** Yi Yang, Handan Tan, Bolin Deng, Hongsong Yu, Guannan Su, Jiayue Hu, Qingfeng Cao, Gangxiang Yuan, Aize Kijlstra, Peizeng Yang

**Affiliations:** 1 The First Affiliated Hospital of Chongqing Medical University, Chongqing Key Laboratory of Ophthalmology and Chongqing Eye Institute, Chongqing, P. R. China; 2 The second hospital of Lanzhou University, Lanzhou, Gansu P. R. China; 3 University Eye Clinic Maastricht, Maastricht, The Netherlands

## Abstract

C-type lectin receptors (CLRs) have been demonstrated to be involved in several autoimmune diseases. The role of CLRs in Behcet’s disease (BD) is unknown and thus was the purpose of this study. A two-stage association study was carried out and a total of 766 BD patients and 1674 healthy controls were recruited. Genotyping of 14 SNPs of 13 genes in CLRs was carried out by iPLEX Gold genotyping or polymerase chain reaction-restriction fragment length polymorphism (PCR-RFLP) assay. The expression of mannose binding lectin 2 (*MBL2*) and killer cell lectin like receptor C4 (*KLRC4*) was measured by Real-time PCR. Significantly increased frequencies of the A allele as well as AA genotype of rs1800450 in *MBL2* (Pc = 2.50 × 10^−6^, OR = 1.494; Pc = 2.24 × 10^−6^,OR = 2.899; respectively) and TT genotype of rs2617170 in *KLRC4* (Pc = 2.53 × 10^−6^, OR = 1.695) and decreased frequencies of GG genotype of rs1800450 (Pc = 1.56 × 10−3, OR = 0.689) and C allele as well as CC genotype of rs2617170 (Pc = 2.05 × 10−9,OR = 0.664; Pc = 1.20 × 10−5, OR = 0.585; respectively) were observed in BD. Two variants, p.Gly54Asp (rs1800450) and p.Asn104Ser (rs2617170) affect *MBL2* and *KLRC4* protein stability and expression. Our study demonstrates that the *MBL2*/rs1800450 and *KLRC4*/rs2617170 are susceptibility factors for BD in a Chinese Han population.

## Introduction

Behcet’s disease (BD) is a well-known multisystem vasculitis, characterized by recurrent uveitis, oral ulcerations, genital ulcerations and typical skin lesions^1^. It often occurs in young adulthood and causes serious disability and significant visual impairment. BD is more frequent among populations along the “silk route” from the Mediterranean Basin to East Asia^2^. Although the pathogenesis of BD is not yet exactly known, it has been hypothesized that autoimmunity and genetic factors are responsible for this disease^3^. Recent studies have implicated that Human Leukocyte Antigen (HLA) and non-*HLA* genes seem to collectively contribute to the genetic background causing this disorder among different populations^4–12^. Most of the non-HLA genes such as TNFAIP3, IL23R,-IL12RB2, IL10, CCR1, STAT4, KLRC4, ERAP1, FUT2, and IL12A have been reported with genome-wide significance whereas NOS3 and JAK1 were reported with study-wide significance.

C-type lectin receptors (CLRs) are a large group of extracellular Metazoan proteins expressed on immune cells that have been classified as pattern recognition receptors (PRRs) which play an important role in the binding of pathogens via their surface carbohydrate structures. CLRs not only play a pivotal role in the process of anti-inflammatory immune responses but also in the maintenance of host immune-homeostasis^13^. Growing evidence suggests that various members of CLRs are associated with severe immune mediated diseases like juvenile idiopathic arthritis (JIA)^14^, type 1 diabetes (T1DM)^15^, systemic lupus erythematosus (SLE)^16^, rheumatoid arthritis (RA)^17^ and multiple sclerosis (MS)^18^. It was recently shown that patients with BD had significantly lower median serum mannose-binding lectin (MBL) levels compared to healthy controls^19^. Whether gene polymorphisms of CLRs are associated with the susceptibility to BD is not yet known and was therefore the purpose of our study. We identified two variants, p.Gly54Asp (rs1800450) in *MBL2* and p.Asn104Ser (rs2617170) in *KLRC4*, to contribute to the risk of developing BD.

## Results

### Clinical characteristics of BD patients

Clinical features, age as well as gender distribution in the recruited BD patients and healthy controls are presented in Table 1. All patients had uveitis. Our BD patient group contained more males than the control group. The genotype frequencies of the 14 SNPs were tested and the results did not deviate from the Hardy-Weinberg equilibrium in the healthy controls.

**Table 1:** Clinical features, age, and sex distribution of BD patients and healthy controls.

Clinical features	total	%
Patients with BD	766	
Mean age ± SD	33.7 ± 8.9	
Male	663	86.6
Female	103	13.4
Uveitis	766	100
Oral ulcer	766	100
Genital ulcer	454	59.3
Skin lesions	605	79
Arthritis	122	15.9
Positive pathergy test	123	16.1
Healthy controls	1674	
Mean age ± SD	39.5 ± 10.7	
Male	938	56
Female	736	44

### Frequency of genotypes and alleles of the examined SNPs in BD versus healthy controls in the first-phase study

The fourteen SNPs were tested in 388 BD cases and 742 healthy controls during the first phase study. The frequencies of *MBL2*/rs1800450 AA genotype (Pc = 0.02, OR = 2.556) and *KLRC4*/rs2617170 TT genotype (Pc = 8.90 × 10^−3^, OR = 1.688) were significantly higher in BD. A significant lower frequency of the *KLRC4*/rs2617170 C allele and CC genotype was also observed (Pc = 1.66 × 10^−4^, OR = 0.661; Pc = 1.02 × 10^−2^, OR = 0.576; respectively) in BD (Table 2). However, we failed to find a significant association between the remaining SNPs and ocular BD (Supplementary Table 1).

**Table 2:** Genotype and allele frequencies of *MBL2* and *KLRC4* polymorphisms in BD and healthy controls.

Gene	SNP	Allele	BD	%	Controls	%	P Value	Pc Value	OR(95%CI)
		Genotype	N		N				
MBL2	rs1800450								
	stage1	A	188	24.2	284	19.1	5 × 10^−3^	0.26	1.351 (1.096–1.665)
		G	588	75.8	1200	80.9			
		AA	32	8.2	25	3.4	4.25 × 10^−4^	0.02	2.556 (1.492–4.308)
		AG	124	32	234	31.5	0.885	NS	1.020 (0.783–1.327)
		GG	232	59.8	483	65.1	0.079	NS	0.797 (0.619–1.027)
	stage2	A	198	26.2	332	17.8	1.31 × 10^−6^	6.70 × 10^−5^	1.637 (1.339–2.002)
		G	558	73.8	1532	82.2			
		AA	26	6.9	21	2.3	4.54 × 10^−5^	2.32 × 10^−3^	3.204 (1.780–5.769)
		AG	146	38.6	290	31.1	0.009	NS	1.391 (1.084–1.785)
		GG	206	54.5	621	66.6	3.72 × 10^−5^	1.90 × 10^−3^	0.6 (0.470–0.766)
	combined	A	386	25.2	616	18.4	4.90 × 10^−8^	2.50 × 10^−6^	1.494 (1.293–1.726)
		G	1146	74.8	2732	81.6			
		AA	58	7.6	46	2.7	4.39 × 10^−8^	2.24 × 10^−6^	2.899 (1.949–4.312)
		AG	270	35.2	524	31.3	5.40 × 10^−2^	NS	1.195 (0.997–1.431)
		GG	438	57.2	1104	65.9	3.06 × 10^−5^	1.56 × 10^−3^	0.689 (0.579–0.821)
KLRC4	rs2617170								
	stage1	C	342	44.1	807	54.4	3.25 × 10^−6^	1.66 × 10^−4^	0.661 (0.555–0.787)
		T	434	55.9	677	45.6			
		CC	79	20.4	228	30.7	2.00 × 10^−4^	1.02 × 10^−2^	0.576 (0.430–0.772)
		CT	184	47.4	351	47.3	0.97	NS	1.005 (0.786–1.285)
		TT	125	32.2	163	22	1.74 × 10^−4^	8.90 × 10^−3^	1.688 (1.283–2.222)
	stage2	C	333	44	1009	54.1	2.89 × 10^−6^	1.47 × 10^−4^	0.667 (0.563–0.791)
		T	423	56	855	45.9			
		CC	77	20.4	281	30.2	3.19 × 10^−4^	1.63 × 10^−2^	0.593 (0.445–0.789)
		CT	179	47.4	447	48	0.84	NS	0.976 (0.768–1.240)
		TT	122	32.3	204	21.9	8.16 × 10^−5^	4.16 × 10^−3^	1.701 (1.304–2.218)
	combined	C	675	44.1	1816	54.2	4.03 × 10^−11^	2.05 × 10^−9^	0.664 (0.588–0.750)
		T	857	55.9	1532	45.8			
		CC	156	20.4	509	30.4	2.35 × 10^−7^	1.20 × 10^−5^	0.585 (0.477-0.718)
		CT	363	47.4	798	47.7	0.897	NS	0.989 (0.833–1.173)
		TT	247	32.2	367	21.9	4.96 × 10^−8^	2.53 × 10^−6^	1.695 (1.401–2.051)

SNP, single-nucleotide polymorphism; BD, Behcet’s disease; OR, odds ratio; NS, not significant; 95% CI, 95% confidence interval; Pc, Bonferroni corrected p value.

### Genotype and allele frequency of the examined SNPs in BD versus healthy controls in the second phase and combined study

To validate the results from the first stage study, a different cohort of patients (378 BD cases and 932 healthy individuals) was recruited for the second stage study. The frequencies of the *MBL2*/rs1800450 A allele and AA genotype in BD patients was significantly higher (Pc = 6.70 × 10^−5^, OR = 1.637; Pc = 2.32 × 10^−3^, OR = 3.204; respectively), whereas a lower frequency of the GG genotype (Pc = 1.90 × 10^−3^, OR = 0.6) was observed in BD compared with controls (Table 2). The *KLRC4*/rs2617170 C allele and CC genotype frequencies were significantly lower in BD compared to controls (Pc = 1.47 × 10^−4^, OR = 0.667; Pc = 1.63 × 10^−2^, OR = 0.593, respectively), while a higher frequency of the TT genotype was detected in BD (Pc = 4.16 × 10^−3^, OR = 1.701) (Table 2). Combination of the data from the two stage studies confirmed that rs1800450 in *MBL2* was correlated with the risk to BD (A allele: Pc = 2.50 × 10^−6^, OR = 1.494; AA genotype: Pc = 2.24 × 10^−6^, OR = 2.899; GG genotype: Pc = 1.56 × 10^−3^, OR = 0.689;) (Table 2), and that rs2617170 in *KLRC4* also contributed to susceptibility of BD (C allele: Pc = 2.05 × 10^−9^, OR = 0.664; CC genotype: Pc = 1.20 × 10^−5^, OR = 0.585; TT genotype: Pc = 2.53 × 10^−6^, OR = 1.695) (Table 2).

### The Influence of *MBL2*/rs1800450 and *KLRC4*/rs2617170 on gene mRNA transcription and cytokine production

In order to find a biological explanation for the association of BD with *MBL2*/rs1800450 and *KLRC4*/rs2617170, the mRNA expression of *MBL2* as well as *KLRC4* of healthy genotyped individuals was measured in their PBMCs. We also evaluated whether different genotypes of *MBL2*/rs1800450 and *KLRC4*/rs2617170 could influence the production of cytokines important in the development of BD such as IFN-γ, IL-6, IL-8, IL-1β, IL-10 and TNF-α. These experiments were performed in healthy individuals to eliminate confounding effects such as the inflammatory status and immunosuppressive drug effects in our BD patient group.

**Figure 1:**
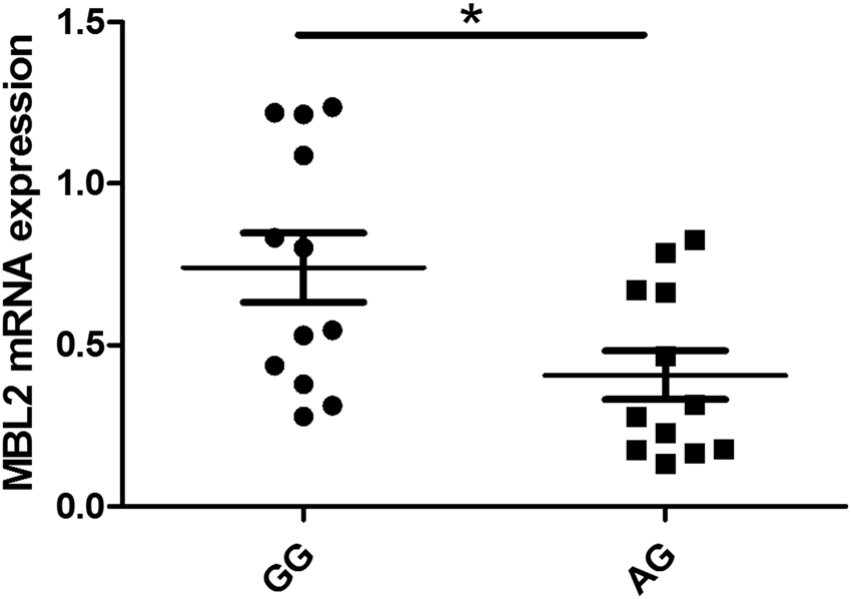
The influence of *MBL2*/rs1800450 genotypes on the expression of *MBL2* by PBMCs. *MBL2* mRNA level in GG individuals of SNP rs1800450 was significantly higher than in AG individuals. Data are shown as mean ± SD. *P = 0.019 (GG/AG = 12).

**Figure 2:**
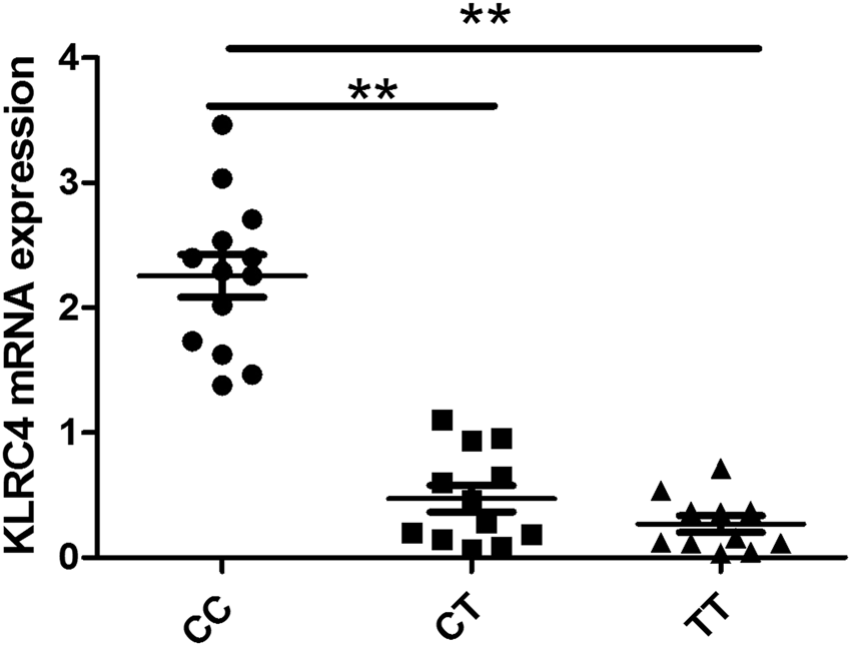
The influence of *KLRC4*/rs2617170 genotypes on the expression of *KLRC4* in PBMCs. *KLRC4* mRNA level in CC individuals of SNP rs2617170 was significantly higher than in CC/CT individuals. Data are shown as mean ± SD. **P < 0.001 (CC = 13, CT = 11, TT = 10).

Real-time PCR data demonstrated that the mRNA level of *MBL2*/rs1800450 in GG carriers was remarkably higher than AG carriers (P = 0.019) (Fig. 1). We did not test AA carriers since the frequency of this genotype is very low (<3–4%). The mRNA expression of *KLRC4*/rs2617170 in CC carriers showed a significant increase compared to CT/TT individuals (Fig. 2, P < 0.001).

**Figure 3:**
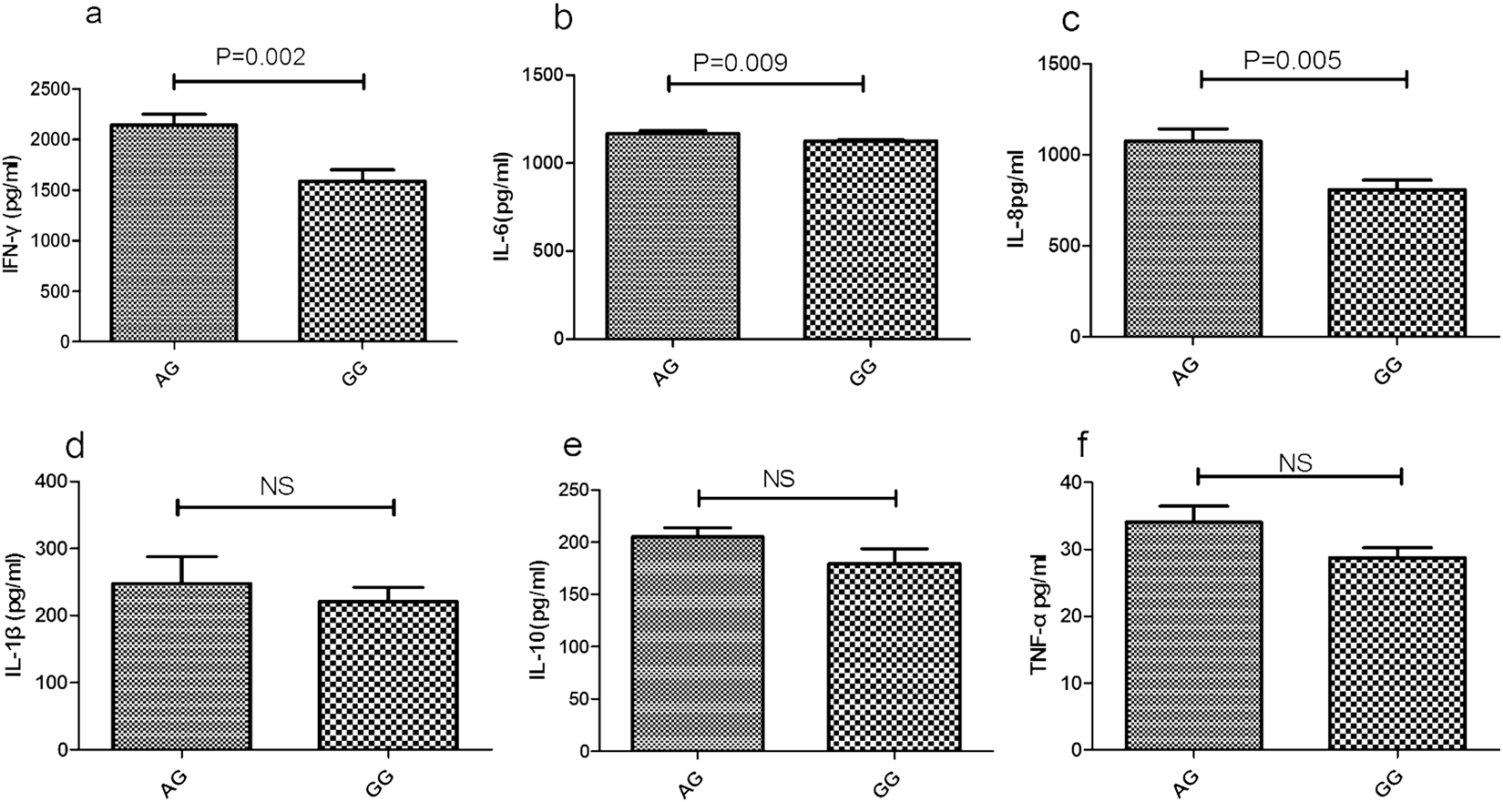
Effect of MBL2 genotype on cytokine production by LPS stimulated PBMCs from healthy genotyped individuals. IFN-γ (**a**), IL-6 (**b**), IL-8(**c**), IL-1β (**d**), IL-10 (**e**) and TNF-α (**f**) were measured by ELISA in the cell culture supernatants. Date expressed as the mean ± SD (AG/GG = 12).

**Figure 4:**
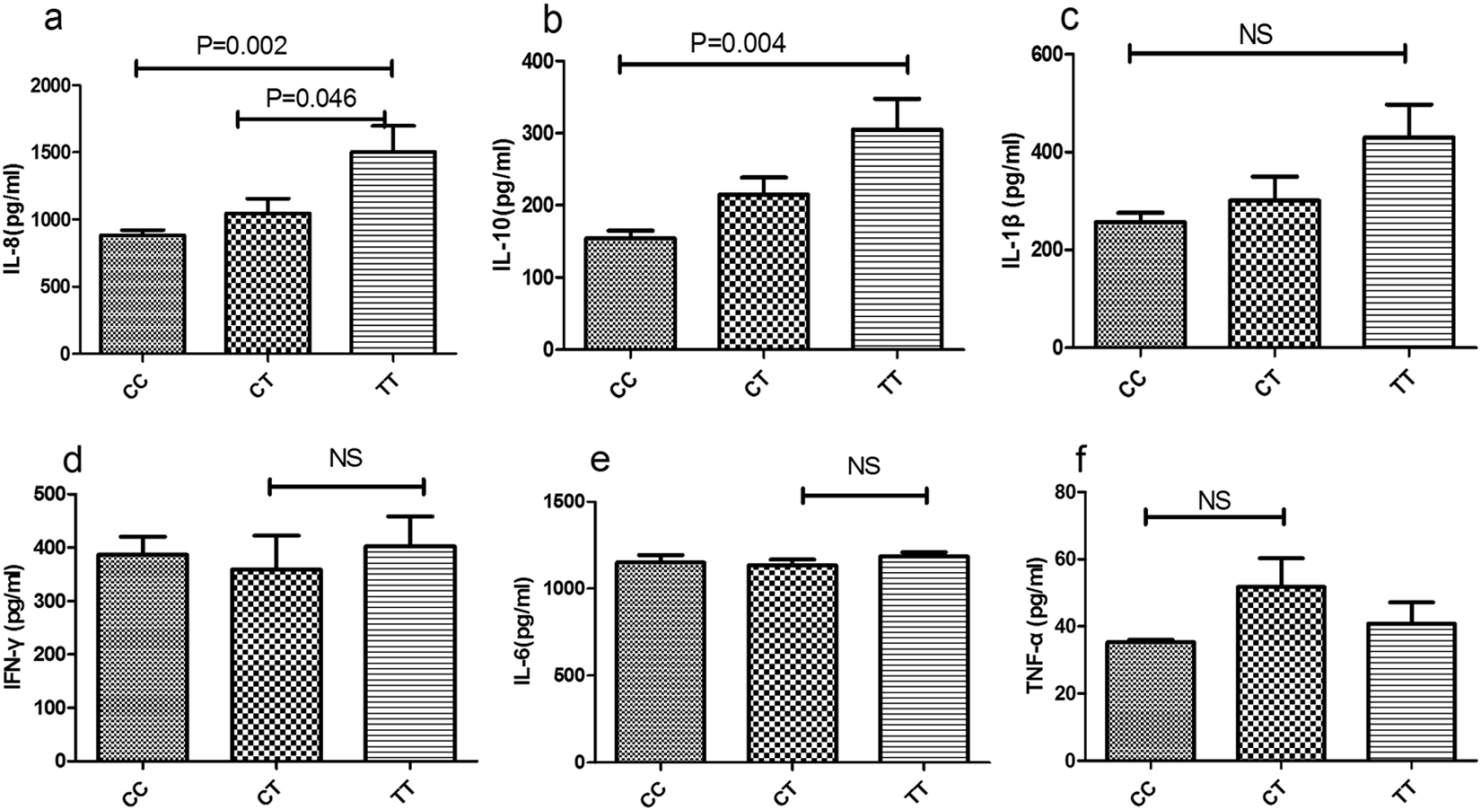
Effect of *KLRC4* genotype on cytokine production by LPS stimulated PBMCs from healthy genotyped individuals. IL-8 (**a**), IL-10 (**b**), IL-1β (**c**), IFN-γ (**d**), IL-6 (**e**) and TNF-α (**f**) were measured by ELISA in the cell culture supernatants. Data are expressed as the mean ± SD (CC = 10, CT = 10, TT = 8).

The effect of *MBL2* and *KLRC4* genotype on cytokine production was tested in LPS treated PBMCs isolated from genotyped healthy controls. ELISA was applied to test the concentration of IFN-γ, IL-6, IL-8, IL-1β, IL-10 as well as TNF-α in the 72 hr cell culture supernatants. LPS stimulated PBMCs from GG genotype *MBL2*/rs1800450 carriers secreted higher amount of INF-γ, IL-6 and IL-8 than AG carriers (P = 0.002; p = 0.009; p = 0.005; respectively) (Fig. 3a,b,c). Compared to CC (P = 0.002; P = 0.004) carriers, an elevated secretion of IL-8 and IL-10 was observed in TT *KLRC4*/rs2617170 (Fig. 4a,b). No effect of the various rs1800450 and rs2617170 genotypes on the release of other cytokines could be detected.(Figs 3d,e,f and 4c,d,e).

## Discussion

In this study we show that gene polymorphisms of *MBL2* encoding rs1800450 and *KLRC4* encoding rs2617170 are associated with BD. Furthermore, the two SNPs were found to affect their gene expression. mRNA expression of MBL2 and *KLRC4* were higher in individuals with the GG(BD-protective) genotype of rs1800450 and CC(BD-protective) genotype of rs2617170 as compared to the other genotype carriers. Additionally, INF-γ, IL-6 and IL-8 production by stimulated PBMCs from GG genotype carriers of rs1800450 and IL-8, IL-10 production by stimulated PBMCs from CC genotype carriers of rs2617170 were increased.

C-type lectin receptors (CLRs), often containing the C-type lectin-like domain (CTLD), are a large family of extracellular proteins^13^. CLRs can bind carbohydrate through CTLD and activate different signaling pathways, which induce the expression of specific cytokines ultimately affecting T cell subtype polarization^20^. Recent findings also showed that CLRs are vital in immune homeostasis, which can induce both pro-inflammatory and anti-inflammatory immune responses^20^. BD is a multifactorial autoinflammatory disease and the interactions between susceptibility genes and environmental factors may affect susceptibility^3^. Several studies suggest that the expression of some members of CLRs are significantly different between BD cases and healthy individuals, such as the increased CD94 expression in BD patients^21^ and decreased mannose-binding lectin (MBL) concentration as compared to healthy controls^18^. Moreover, previous reports showed that CLRs are involved in the development of certain autoimmune diseases such as JIA, T1MD, SLE, RA, MS^14–18^. Based on these studies, we assumed that CLR genetic polymorphisms might also be associated with BD. To validate this hypothesis, we examined the association of polymorphisms of CLRs in BD patients and found a strong association between 2 SNPs, rs1800450 in the *MBL2* gene and rs2617170 in *KLRC4*, with BD in a Chinese Han population. The fact that CLRs play vital roles in the innate immune response against microbial pathogens strengthens the view that BD is caused by an aberrant response against environmental stimuli^20^.

*MBL2* belongs to the C-type collectin family, and plays a potential role in innate immunity. Many studies showed that a low or high serum MBL level is involved in several immune mediated diseases (e.g., RA, Crohn’s disease, Sjögren disease and diabetic retinopathy^22–24^). Immune defense function of MBL is associated with its serum level and oligomeric type^23^. Five SNPs of the *MBL2* gene, including three structure variants, codon 52 (rs5030737), 54 (rs1800450), 57 (rs1800451) and two promoter variants, −550 (rs11003125) and −221(rs7096206) are thought to be responsible for reducing MBL2 serum levels and influencing the formation as well as the stability of oligomeric MBL2^25–27^. However, others didn’t find any association between genetic polymorphisms of *MBL2* and BD susceptibility^28^. In our study, we confirmed that *MBL2* is a predisposing gene for BD in a Chinese Han population. Sample selection bias and different genetic backgrounds may explain the observed discrepancy between studies. We did not measure *MBL2* levels in serum of our BD patients or controls, since the patients were often treated with immunosuppressive drugs, which may influence the serum concentration of *MBL2*. Further studies are needed to address this issue.

NKG2F encoded by the *KLRC4* gene is a recently described member of the NKG2 family receptors, and its function has not been examined in detail^29^. This receptor can activate NK cells following the binding with its ligand DAP12^30^. A recent GWAS has shown that rs2617170 of *KLRC4* is associated with BD in Turkish and Japanese patients^31^. However, it has not yet been reported in the Chinese population. Our results indicate that only the rs2617170 association (C allele: Pc = 2.05 × 10^−9^, OR = 0.664; CC genotype: Pc = 1.20 × 10^−5^, OR = 0.585) exceeds the threshold for genome-wide significance (P < 5e-08), Our study confirms the results of a previous GWAS regarding the association of *KLRC4*/rs2617170 with BD^32^. Interestingly, the C allele of rs2617170 was associated with disease risk in this GWAS study^31^, whereas the C allele had a higher frequency in the controls as compared to the BD patients in our study, and would therefore seem to be associated with disease protection. On the other hand, Dixon *et al* ^32^. have reported that rs2617170 is a significant eQTL for *KLRC4* expression, and the C allele is associated with reduced *KLRC4* gene expression. This is in contrast with our findings where we showed that the C allele is associated with higher gene expression. The reasons for these discrepancies may be due to different ancestral backgrounds of the subjects investigated and this issue clearly deserves further study. Until now, the role of *KLRC4* in BD has not received much attention. It has been demonstrated that the stimulation of IL-2 and IL-15 led to an up-regulation of *KLRC4* on NK cells^30^. Other groups have reported that IL-15 levels were elevated in serum, cerebrospinal fluid, and aqueous humor from patients with BD^33–35^. Further experiments are needed to unravel the functional role of *KLRC4* variants on BD pathogenesis. It is interesting to point out that we observed a lower frequency of CC (30.4%) and a higher frequency of CT of rs2617170 in our healthy control group (47.7%) as compared to data reported in the Asian population as shown in the NCBI Resource (42.2% and 35.6%, respectively). However, our results are similar to a previous report on rs2617170 genotype frequencies^36^ in a Chinese Han population (27.4% and 55.8%, respectively), indicating heterogeneity between Asian populations.

Our study has a number of limitations. Firstly, since we only chose the loci with known associations between CLRs and various autoimmune or auto inflammatory diseases, it cannot be excluded that other SNPs in CLRs may have an association with BD. Detailed sequence analysis should be carried out to investigate the potential involvement of other rare variants of these factors in BD development. Secondly, our BD patients were Chinese Han patients recruited from an ophthalmic department and all had uveitis. Not all patients with BD have uveitis and depending on their complaints will see different medical departments. Further studies including BD patients from other medical departments (e.g., dermatology, rheumatology, stomatology) and other populations are therefore required to confirm our results and to investigate whether the observed associations are not only confined to the subpopulation of BD patients with uveitis. Due to sample size we also did not investigate whether subgrouping of our patients according to clinical features had an effect on the CLR gene associations. Last but not least, although our study identified rs1800450 of *MBL2* and rs2617170 of *KLRC4* as possible risk factors contributing to the susceptibility for BD, the exact mechanism how these variants affect the disease pathogenesis are not yet exactly clarified and await further study.

In summary, our study confirmed that *MBL2*/rs1800450 and *KLRC4*/rs2617170 polymorphisms affect disease susceptibility in the Chinese Han population. Further studies are needed to reveal the crucial role of the CLRs pathways in the pathogenesis of BD.

## Materials and Methods

### Study population

All BD uveitis patients (n = 766) and healthy individuals (n = 1674) included in the present study (n = 2440) were ethnic Han Chinese, recruited from the First Affiliated Hospital of Chongqing Medical University from May 2008 to August 2015. The diagnostic criteria of BD strictly followed the International Study Group for BD^37^. Controls were matched for age, geographic origin and ethnicity with BD patients. A case-control study including two phases was performed. In the first phase, 388 BD patients and 742 healthy individuals were included. and in the second phase, 378 BD cases and 932 controls were recruited. The major clinical symptoms in the recruited BD cases are clarified in Table 1. This study was conducted under the approval of the Clinical Research Ethics Committee of the First Affiliated Hospital of Chongqing Medical University (Permit Number: 2009-201008). All the procedures complied with the tenets of the Declaration of Helsinki. Informed written consent was provided by all patients and controls.

### Single nucleotide polymorphisms (SNPs) selection

SNPs were chosen from previous studies on the correlation between CLRs and various autoimmune or auto-inflammatory diseases^31, 38–46^.Minor allele frequency (MAF) and linkage disequilibrium (LD) were tested by HaploView 4.2 software based on the data of Han Chinese in the HapMap database (MAF > 0.05 as well as an r^2^-value of LD < 0.8). Based on this analysis, we selected a total of 14 SNPs, including 2 SNPs (rs1800450^38^, rs7096206^39^) in *MBL2*, one SNPs (911887^40^) in *SFTPD*, one SNP (rs1323461^41^) in *CLEC12A*, one SNP (rs2377422^42^) in *CLEC4A*, one SNP (3764022^43^) in *CLEC2D*, one SNP (rs2287886^44^) in *CD209*, one SNPs (rs4763879^15^) in *CD69*, one SNP (rs2302489^45^) in *KLRD1*, one SNP (rs2734440^17^) in *KLRC1*, one SNP (rs2255336^17^) in *KLRK1*, one SNP (rs2617170^31^) in *KLRC4*, one SNP (rs4763655^18^) in *KLRB1* and one SNP (rs1121401^46^) in *KLRG1*. *CLEC16A* ^47^ and selectins^48^ were excluded from this study because they have been reported previously by our team.

### Genomic DNA Extraction and Genotyping

Genomic DNA extraction from peripheral blood cells was performed using the QIAamp DNA Blood Mini Kit (Qiagen, Valencia, California, USA), according to instructions of the manufacturer. The concentration and quality of DNA were analyzed with a Nanodrop 2000 (Thermo Fisher Scientific, Wilmington, DE, USA) and then the DNA samples were standardized and kept at −20 °C until used.

In the first stage, all SNPs in our study (except rs1800450) were genotyped by the MassARRAY platform (Sequenom, USA) and iPLEX Gold Assay. The PCR reaction was carried out by the Gene Amp PCR System 9700 instrument (ABI, Foster City, CA, USA). MassARRAY Assay design software was used to design the primers (Table 3). Experimental data were analyzed through SpectroTYPER software (version 4.0; Sequenom). Rs2617170 in the second stage was performed by the TaqManH SNP Genotyping Assay in the 7500 Real-Time PCR system (Applied Biosystems, USA). The results were examined through TaqManH Genotyper Software. Rs1800450 was genotyped by the PCR-RFLP method.

**Table 3:** Primers applied in the analysis of iPLEX Gold genotyping in the CLR related genes.

SNP_ID	2nd-PCRP	1st-PCRP	UEP_SEQ
MBL2			
rs7096206	ACGTTGGATGACCTGGGTTTCCACTCATTC	ACGTTGGATGTTCATCTGTGCCTAGACACC	TGTTCTCACTGCCAC
CD69			
rs4763879	ACGTTGGATGTGTTGCATGTATCAGTTGTC	ACGTTGGATGTGCAAGAATGCTCCTAGCAG	TTGTCTTATTTTGAATTGCTGAG
SFTPD			
rs911887	ACGTTGGATGCCCTGTATACAGACTTCTCC	ACGTTGGATGAAAGGCAGAGGTGGTATCGC	aaccGACTTCTCCATTGCTTGCGCC
KLRD1			
rs2302489	ACGTTGGATGAGCTGAGCTGGAGATTAAAG	ACGTTGGATGGAGGCTTGTGATTCTACTGC	ttAGTATGAAGAAATTTAGCAAAAA
KLRC4			
rs2617170	ACGTTGGATGTTTTGCATCCCTTTAGAGAC	ACGTTGGATGAGGTATTGGAGTACTGGAGC	gttcgGCATTCTTCTATTCAGGGAAAAA
CD209			
rs2287886	ACGTTGGATGATTCTTGAAAGATCCGGCCC	ACGTTGGATGTCCCACCCTGTGATCTTTAC	TCTGATGCTTTCCACTAG
KLRB1			
rs4763655	ACGTTGGATGCCTGACCCCAGTGTATTATG	ACGTTGGATGTCTCACATTAGGATGCTCAC	ttTTCTATCTCCTCAGGGC
CLEC12A			
rs1323461	ACGTTGGATGGAGTTTAGGCACTCAGATCC	ACGTTGGATGTTTCTGACCCACACTCCTAC	ggccGATCCCTGCATACTCAT
KLRK1			
rs2255336	ACGTTGGATGGCAATCTACTTCTCTGTTGTC	ACGTTGGATGTTTCTGCTGCTTCATCGCTG	AGGAATACAGCACTCCATATTG
CLEC4A			
rs2377422	ACGTTGGATGCCTCCCTACCTTTCATTTGC	ACGTTGGATGGGAAGAGGACTAAGTAACCC	ATTTCACTAAAACCATCCCTAAA
CLEC2D			
rs3764022	ACGTTGGATGCCTCTAGTGAAAAGCGAAGG	ACGTTGGATGAAGCGCCAAATTAAGGTAGC	TTTCAATAATTTTTTCCAGGTTGT
KLRC1			
rs2734440	ACGTTGGATGAGGACAATGGCCACAATGAC	ACGTTGGATGCAGCCCATGAAGATGTATAG	CATATTTGCAAACATATAAACCTATA
KLRG1			
rs1121401	ACGTTGGATGAGTGACCTATGAACAATGCC	ACGTTGGATGATACCTGTAGGTTGTATCCC	AATAGTATAACAAAAGTGAAACTG

### Cell isolation and culture

Ficoll-Hypaque density-gradient centrifugation was used to separate Peripheral blood mononuclear cells (PBMC) from fresh venous blood of healthy male controls. PBMCs were seeded into 24-well culture plates (1 × 10^6^ cells/well) in medium RPMI 1640 (supplemented with 10% fetal calf serum, 100 U/ml penicillin and 100 μg/ml streptomycin). To test the production of cytokine IFN-γ, IL-6, IL-8, IL-1β, IL-10 and TNF-α, PBMCs were stimulated with 100 ng/ml lipopolysaccharide (LPS, 100 ng/ml; Sigma, Missouri, USA) for 24 h.

### Real-time PCR

Total RNA extraction from PBMCs was performed using the TRIzol (Invitrogen, San Diego, California, USA) method. RNA was reverse transcribed into cDNA with a Takara transcriptase kit (Takara, Dalian, China). The assays were carried out on an ABI 7500 real-time system with the following primers (*KLRC4*: 5′-GGAATGACAAGACATATCACTG-3′and 5′-GTCAGTTGAATACTACACAGACT-3′; *MBL2*: GCAAACAGAAATGGCACGTAT and AGAGGCCTGGAACTTGACA). The expression level was measured by the 2^−ΔΔCt^ method.

### Measurement of cytokines by ELISA

The concentration of IFN-γ, IL-6, IL-8, IL-1β, IL-10 and TNF-α in the supernatants of PBMCs were analyzed through using the human Duoset ELISA development kit (R&D Systems, Minneapolis, Minnesota, USA).

### Statistical analysis

The differences between BD cases and healthy individuals with regard to allele and genotype frequencies were analyzed by the chi-square (χ2) test with SPSS17.0 statistical software package (version17.0, SPSS, Chicago, IL). Hardy-Weinberg equilibrium was examined by the SHEsis website.

For multiple comparisons, the Bonferroni correction was used to adjust P values to corrected P values (Pc) according to the number of performed analyses. A p_c_ < 0.05 was viewed as significant. Expression of *KLRC4*, *MBL2* and cytokine levels among three genotype groups was tested by the non-parametric Mann-Whitney test or student t test, with P < 0.05 (Two-tailed) taken as being statistically significant.

## Electronic supplementary material

The frequency of genotypes and alleles of CLRs except MBL2 and KLRC4 in BD versus healthy controls.
