# Supplementary material for: Genetic polymorphisms of C-type lectin receptors in Behcet’s disease in a Chinese Han population
Source: Sci Rep. 2017 Jul 13;7:5348. doi: 10.1038/s41598-017-05877-x (PMC5509750; doi:10.1038/s41598-017-05877-x)
Supplement: Supplementary file 1 — The frequency of genotypes and alleles of CLRs except MBL2 and KLRC4 in BD versus healthy controls. [file 41598_2017_5877_MOESM1_ESM.doc]

**Genetic polymorphisms of C-type lectin receptors in Behcet’s disease in a Chinese Han population**

**Yi Yang^1, 2^, Handan Tan^1^, Bolin Deng^1^, Hongsong Yu^1^,** **Guannan Su^1^, Jiayue Hu^1^, Qingfeng Cao^1^, Gangxiang Yuan^1^, Aize Kijlstra^3^, Peizeng Yang^1^**

1 The First Affiliated Hospital of Chongqing Medical University, Chongqing Key Laboratory of Ophthalmology and Chongqing Eye Institute, Chongqing, P. R. China

2 The second hospital of Lanzhou University, Lanzhou, Gansu, P. R. China

3 University Eye Clinic Maastricht, Maastricht, The Netherlands

**Supplemental Table 1 The frequency of genotypes and alleles of CLRs except MBL2 and KLRC4 in BD versus healthy controls.**

| Gene |  | | SNP | Allele | BD |  | Controls |  | P Value | Pc Value | OR(95%CI) |
| --- | --- | --- | --- | --- | --- | --- | --- | --- | --- | --- | --- |
|  |  |  | | Genotype | N | % | N | % |  |  |  |
| CD69 |  | rs4763879 | | A | 346 | 44.7 | 660 | 44.4 | 0.874 | NS | 1.014(0.852-1.208) |
|  |  |  | | AA | 85 | 22 | 158 | 21.2 | 0.778 | NS | 1.044(0.775-1.406) |
|  |  |  | | AG | 176 | 45.5 | 344 | 46.2 | 0.808 | NS | 0.970(0.758-1.241) |
|  |  |  | | GG | 126 | 32.6 | 242 | 32.5 | 0.992 | NS | 1.001(0.770-1.302) |
| MBL2 |  | rs7096206 | | C | 621 | 83.9 | 1269 | 84.9 | 0.529 | NS | 0.925（0.726-1.179） |
|  |  |  | | CC | 261 | 70.5 | 539 | 72.2 | 0.573 | NS | 0.924（0.702-1.216） |
|  |  |  | | CG | 99 | 26.8 | 191 | 25.6 | 0.67 | NS | 1.063（0.801-1.411） |
|  |  |  | | GG | 10 | 2.7 | 17 | 2.3 | 0.662 | NS | 1.193（0.541-2.631） |
| SFTPD |  | rs911887 | | C | 263 | 35.3 | 541 | 36.6 | 0.577 | NS | 0.949(0.790-1.141) |
|  |  |  | | CC | 42 | 11.3 | 104 | 14.1 | 0.198 | NS | 0.778(0.531-1.141) |
|  |  |  | | CT | 179 | 48.1 | 333 | 45 | 0.325 | NS | 1.134(0.883-1.455) |
|  |  |  | | TT | 151 | 40.6 | 303 | 40.9 | 0.91 | NS | 0.985(0.765-1.270) |
| KLRD1 |  | rs2302489 | | A | 364 | 49.3 | 796 | 53.9 | 0.044 | NS | 0.834（0.699-0.995） |
|  |  |  | | AA | 85 | 23 | 211 | 28.6 | 0.05 | NS | 0.749（0.560-1.001） |
|  |  |  | | AT | 194 | 52.6 | 374 | 50.6 | 0.537 | NS | 1.082（0.842-1.389） |
|  |  |  | | GG | 69 | 18.2 | 153 | 21 | 0.277 | NS | 0.839(0.612-1.151) |
| KLRK1 |  | rs2255336 | | C | 633 | 82.9 | 131 | 17.1 | 0.018 | NS | 1.314(1.048-1.647) |
|  |  |  | | CC | 261 | 68.3 | 457 | 62.4 | 0.051 | NS | 1.298(0.998-1.687) |
|  |  |  | | CT | 111 | 29.1 | 237 | 32.4 | 0.256 | NS | 0.855(0.653-1.120) |
|  |  |  | | TT | 10 | 2.6 | 38 | 5.2 | 0.256 | NS | 0.855(0.653-1.120) |
| CLEC12A |  | rs1323461 | | A | 175 | 23 | 356 | 24.5 | 0.446 | NS | 0.923(0.750-1.135) |
|  |  |  | | AA | 20 | 5.3 | 47 | 6.5 | 0.426 | NS | 0.804(0.469-1.377) |
|  |  |  | | AC | 135 | 35.5 | 262 | 36 | 0.866 | NS | 0.978(0.755-1.267) |
|  |  |  | | CC | 225 | 59.2 | 418 | 57.5 | 0.583 | NS | 1.073(0.834-1.381) |
| CD209 |  | rs2287886 | | A | 498 | 65.4 | 986 | 67.4 | 0.911 | NS | 0.911(0.757-1.096) |
|  |  |  | | AA | 162 | 42.5 | 322 | 44 | 0.625 | NS | 0.940(0.732-1.207) |
|  |  |  | | AG | 174 | 45.7 | 342 | 46.8 | 0.723 | NS | 0.956(0.746-1.226) |
|  |  |  | | GG | 45 | 11.8 | 67 | 9.2 | 0.116 | NS | 1.331(0.892-1.986) |
| CLEC4A |  | rs2377422 | | C | 431 | 56.6 | 767 | 52.5 | 0.7 | NS | 1.177(0.987-1.404) |
|  |  |  | | CC | 120 | 31.5 | 198 | 27.1 | 0.122 | NS | 1.238(0.944-1.623) |
|  |  |  | | CT | 191 | 50.1 | 371 | 50.8 | 0.876 | NS | 0.981(0.766-1.256) |
|  |  |  | | TT | 70 | 18.4 | 162 | 22.2 | 0.14 | NS | 0.791(0.578-1.081) |
| KLRC1 |  | rs2734440 | | C | 299 | 39.2 | 582 | 40 | 0.737 | NS | 0.970(0.811-1.160) |
|  |  |  | | CC | 63 | 16.5 | 117 | 16.1 | 0.842 | NS | 1.035(0.740-1.446) |
|  |  |  | | CT | 173 | 45.4 | 348 | 47.8 | 0.448 | NS | 0.908(0.708-1.165) |
|  |  |  | | TT | 145 | 38.1 | 263 | 36.1 | 0.526 | NS | 1.086(0.841-1.404) |
| CLEC2D |  | rs3764022 | | C | 481 | 61.7 | 908 | 61.2 | 0.823 | NS | 1.020(0.854-1.219) |
|  |  |  | | CC | 152 | 39 | 278 | 37.5 | 0.619 | NS | 1.066(0.828-1.371) |
|  |  |  | | CG | 177 | 45.4 | 352 | 47.4 | 0.51 | NS | 0.921(0.720-1.177) |
|  |  |  | | GG | 61 | 15.6 | 112 | 15.1 | 0.808 | NS | 1.043(0.743-1.464) |
| KLRB1 |  | rs4763655 | | A | 362 | 47.8 | 641 | 48.1 | 0.872 | NS | 0.985(0.824-1.178) |
|  |  |  | | AA | 87 | 23 | 165 | 24.8 | 0.509 | NS | 0.905(0.672-1.218) |
|  |  |  | | AG | 188 | 49.6 | 311 | 46.7 | 0.366 | NS | 1.124(0.873-1.446) |
|  |  |  | | GG | 104 | 27.4 | 190 | 28.5 | 0.707 | NS | 0.947(0.715-1.255) |
| KLRG1 |  | rs1121401 | | A | 369 | 53.3 | 720 | 51.1 | 0.33 | NS | 1.095(0.912-1.314) |
|  |  |  | | AA | 93 | 26.9 | 175 | 24.8 | 0.472 | NS | 1.113(0.831-1.492) |
|  |  |  | | AT | 183 | 52.9 | 370 | 52.5 | 0.901 | NS | 1.016(0.786-1.315) |
|  |  |  | | TT | 70 | 20.2 | 160 | 22.7 | 0.364 | NS | 0.864(0.630-1.185) |

SNP, single-nucleotide polymorphism; BD, Behcet’s disease; OR, odds ratio; NS, not significant; 95 % CI, 95 % confidence interval; Pc, Bonferroni corrected p value
